# White matter abnormalities in active elite adult rugby players

**DOI:** 10.1093/braincomms/fcab133

**Published:** 2021-07-19

**Authors:** Karl A Zimmerman, Etienne Laverse, Ravjeet Samra, Maria Yanez Lopez, Amy E Jolly, Niall J Bourke, Neil S N Graham, Maneesh C Patel, John Hardy, Simon Kemp, Huw R Morris, David J Sharp

**Affiliations:** 1 Computational, Cognitive and Clinical Neuroimaging Laboratory, Division of Brain Sciences, Hammersmith Hospital, Imperial College London, London W12 0NN, UK; 2 Care Research & Technology Centre, UK Dementia Research Institute, London W12 0BZ, UK; 3 Department of Clinical and Movement Neuroscience, University College London, London NW3 2PF, UK; 4 Centre for the Developing Brain, School of Biomedical Engineering and Imaging Sciences, King’s College London, London SE1 7EH, UK; 5 Imaging Department, Imperial College Healthcare NHS Trust, Charing Cross Hospital, London W6 8RF, UK; 6 Department of Neurodegenerative Disease, Reta Lila Weston Laboratories, Queen Square Genomics, UCL Dementia Research Institute, London WC1N 3BG, UK; 7 Rugby Football Union, Twickenham, London TW2 7BA, UK; 8 Faculty of Epidemiology and Public Health, London School of Hygiene and Tropical Medicine, London WC1E 7HT, UK; 9 The Royal British Legion Centre for Blast Injury Studies, Imperial College London SW7 2AZ, UK

**Keywords:** concussion, TBI, mTBI, imaging, DTI

## Abstract

The recognition, diagnosis and management of mild traumatic brain injuries are difficult and confusing. It is unclear how the severity and number of injuries sustained relate to brain injuries, such as diffuse axonal injury, diffuse vascular injury and progressive neurodegeneration. Advances in neuroimaging techniques enable the investigation of neuropathologies associated with acute and long-term effects of injury. Head injuries are the most commonly reported injury seen during professional rugby. There is increased vigilance for the immediate effects of these injuries in matches, but there has been surprisingly little research investigating the longer-term effects of rugby participation. Here, we present a longitudinal observational study investigating the relationship of exposure to rugby participation and sub-acute head injuries in professional adult male and female rugby union and league players using advanced MRI. Diffusion tensor imaging and susceptibility weighted imaging was used to assess white matter structure and evidence of axonal and diffuse vascular injury. We also studied changes in brain structure over time using Jacobian Determinant statistics extracted from serial volumetric imaging. We tested 41 male and 3 female adult elite rugby players, of whom 21 attended study visits after a head injury, alongside 32 non-sporting controls, 15 non-collision-sport athletic controls and 16 longitudinally assessed controls. Eighteen rugby players participated in the longitudinal arm of the study, with a second visit at least 6 months after their first scan. Neuroimaging evidence of either axonal injury or diffuse vascular injury was present in 23% (10/44) of players. In the non-acutely injured group of rugby players, abnormalities of fractional anisotropy and other diffusion measures were seen. In contrast, non-collision-sport athletic controls were not classified as showing abnormalities. A group level contrast also showed evidence of sub-acute injury using diffusion tensor imaging in rugby players. Examination of longitudinal imaging revealed unexpected reductions in white matter volume in the elite rugby players studied. These changes were not related to self-reported head injury history or neuropsychological test scores and might indicate excess neurodegeneration in white matter tracts affected by injury. Taken together, our findings suggest an association of participation in elite adult rugby with changes in brain structure. Further well-designed large-scale studies are needed to understand the impact of both repeated sports-related head impacts and head injuries on brain structure, and to clarify whether the abnormalities we have observed are related to an increased risk of neurodegenerative disease and impaired neurocognitive function following elite rugby participation.

## Teaser

Using advanced neuroimaging techniques to study a group of professional rugby players, Zimmerman et al. report evidence of both cross-sectional and longitudinal imaging abnormalities in the white matter. Their findings suggest an association of participation in elite adult rugby with changes in brain structure.

## Graphical Abstract

**Graphical Abstract:**
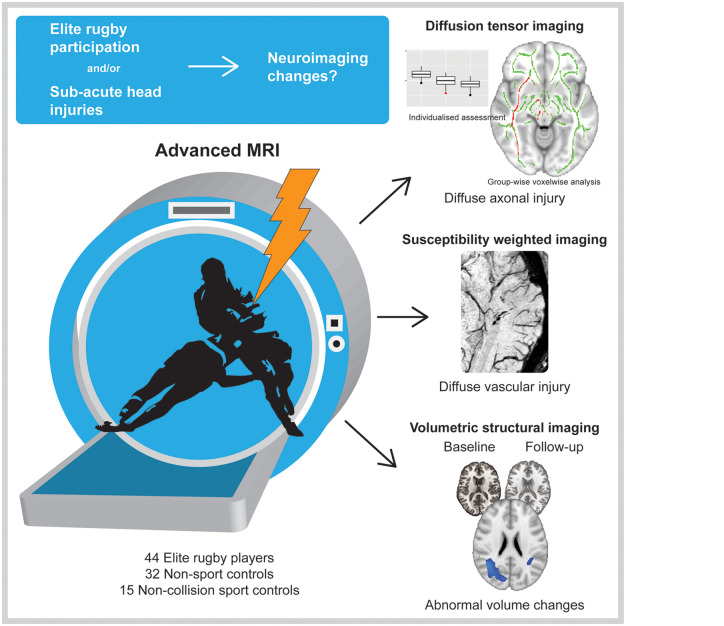

## Introduction

Mild traumatic brain injuries (mTBIs) are common, with a global incidence of 45–54 million per year.^1^ Sport-related mTBIs account for 15–20% of these injuries requiring treatment in the USA.^2^^,^^3^ They are often considered relatively harmless. However, symptoms can persist after injuries that appear relatively trivial^4–6^ and animal models show persistent brain injury after mTBI.^7^ Some types of neuropathology, such as diffuse axonal injury (DAI), are under-recognised by conventional diagnostic imaging and repeated mTBI has been associated with neurodegenerative diseases, such as chronic traumatic encephalopathy (CTE).^8–11^ Hence, detailed investigation of the long-term effects of repeated sports mTBI on brain health is important.

Rugby is a high-intensity collision team sport played in over 120 countries by over 9 million people^12^ and is split into Rugby Union and Rugby League. In professional rugby union in the UK, mTBI is the most common reported match injury (∼20% of injuries) with an incidence of 20.4 per 1000 h.^13^ In Australia, the rate of mTBI in the National Rugby League (NRL) was estimated to be 14.8 per 1000 player match hours.^14^ Despite relatively high rates of head injury and an increasing focus on prevention in the form of law variations^15^ and player preparation,^16^ there has been relatively little research investigating the long-term effects of rugby participation. Previous work in retired rugby players has found evidence of a limited effect of higher self-reported rates of depression and mild cognitive impairment compared to controls.^17–19^ However, rates of dementia in the retired rugby player population are not necessarily different from control levels.^20^ CTE has recently been reported in a small number of ex-players: two in rugby league^21^ and four in rugby union.^22^^,^^23^ Therefore, further research is needed to understand whether exposure to rugby participation and head injuries experienced during a rugby career are sufficient to lead to lasting brain injury.

Neuroimaging is key to the investigation of traumatic brain injury (TBI). Standard CT and MRI often appear normal after mTBI.^5^ However, white matter injuries can be more sensitively identified using advanced magnetic resonance imaging (MRI) sequences, such as diffusion MRI and susceptibility weighted imaging (SWI).^24–26^ DAI causes many of the long-term effects of TBI. It can be sensitively identified by diffusion MRI. Abnormalities in the directionality of water diffusion, most commonly quantified using changes in fractional anisotropy (FA), provide information about the location and severity of DAI. White matter FA increases acutely and subsequently reduces in the presence of longer-term damage.^27–29^ FA and other diffusion metrics provide: (i) a validated measure of white matter integrity after TBI in animals models of TBI e.g. Mac Donald et al.^30^; (ii) increased sensitivity to DAI compared to conventional MRI^24^; and (iii) information that helps predict long-term clinical outcome after TBI,^31^ including the type of post-traumatic problems that develop.^26^^,^^32–34^

Diffusion abnormalities have been demonstrated in a range of sporting mTBI types, however, the direction of change is inconsistent. In mixed sport cohorts, ice hockey players and American Footballers, FA has been shown to increase acutely after a sports mTBI.^35–38^ This elevation has been attributed to increased anisotropy caused by cerebral oedema.^27^^,^^39^ In several studies, these changes persist in the chronic stage,^37^^,^^40^^,^^41^ particularly in cohorts of younger athletes. In others, including studies of boxers, FA is observed to be decreased relative to controls in the chronic stages after a sports mTBI,^42–45^ similar to changes observed in non-sport mTBI and moderate-severe TBI. Several studies do not find abnormalities in FA as a result of sports mTBI.^46–49^ Few studies investigate solely a population of rugby players with diffusion tensor imaging (DTI). In 73 non-injured female varsity^50^ and 11 retired rugby league players,^51^ evidence of reduced FA as well as elevated diffusivity was observed in both studies.

SWI provides additional information about the presence of diffuse vascular injury (DVI).^52^ Gross intracerebral haemorrhage identifiable on CT is relatively uncommon following sports mTBI. However, more subtle DVI can occur and may be missed on conventional imaging. DVI produces small peri-venular haemorrhages that are seen as petechial microhaemorrhages on SWI. Post-traumatic microhaemorrhages are associated with more cognitive impairment and worse clinical outcomes.^53^^,^^54^ However, studies utilizing SWI in a sporting context are very limited. The majority of studies in boxing, ice hockey and American Football have found little to no evidence of abnormal microhaemorrhage prevalence in athletes.^55–58^ Currently, there exist no studies of rugby players using SWI.

In addition to the acute effects of sport mTBI, repeated head impacts can lead to progressive neurodegeneration.^7^^,^^10^^,^^59^ Therefore, an important question is to what extent different sports accelerate neurodegeneration and increase the risk of dementia. Most recent research has focussed on sports, such as American Football and boxing, where the long-term risks of CTE and other types of dementia have been clearly demonstrated.^10^^,^^11^^,^^22^^,^^60^ The neurodegenerative risks of other sports are less clear and the prevalence of sports related dementia is generally uncertain as is the dose of head injury exposure needed to accelerate neurodegeneration. Recent work has demonstrated a surprisingly large increase in the risk of dementia, in particular Alzheimer’s disease, in deceased association football players.^61^ In rugby, CTE pathology has been reported in small case studies of ex-rugby players,^22^^,^^23^ but the prevalence and consequences of neurodegenerative pathology in rugby players are unclear.

The presence of neurodegeneration can be studied using serial MRI. Brain atrophy is a common end-point of various types of neurodegeneration. This is routinely used in the assessment of dementia and can be sensitively measured using longitudinal brain scanning. Serial structural imaging can be used to generate metrics of volume change over time, such as the Jacobian Determinant (JD).^62^ White matter volume changes are highly dependent on age. Volumes increase in children and young adults, before declining in older age.^63^^,^^64^ The normal development and ageing of the white matter can be affected by TBI. Atrophy is increased after single moderate-severe TBI,^65^ is more prominent in the depths of sulci where CTE pathology occurs^66^ and is associated with poorer clinical outcomes.^67–70^ In other neurodegenerative diseases, atrophy measured by MRI has been histologically validated at post-mortem.^71^ Hence, serial MRI provides a method of sensitively assessing whether neurodegeneration is taking place and mapping its spatial pattern, which may give an indication of the underlying pathological process.

Here, we report a longitudinal observational study of 41 male and 3 female elite rugby union and league players using repeated diffusion, susceptibility weighted and volumetric MRI to measure DAI, DVI and brain atrophy, respectively. We test the hypotheses that participation in elite rugby will be associated with: (i) evidence of DAI measured by diffusion MRI; (ii) evidence of DVI measured by SWI; and (iii) evidence of abnormal white matter tract development and/or increased brain atrophy, measured by longitudinal changes in brain volume. To investigate the acute and long-term impact of rugby participation, players were recruited with or without a recent history of mTBI. Players were compared against sporting and non-sporting controls to investigate whether imaging abnormalities were associated with participation in high intensity sporting activity.

## Materials and methods

### Subjects

**Figure 1:**
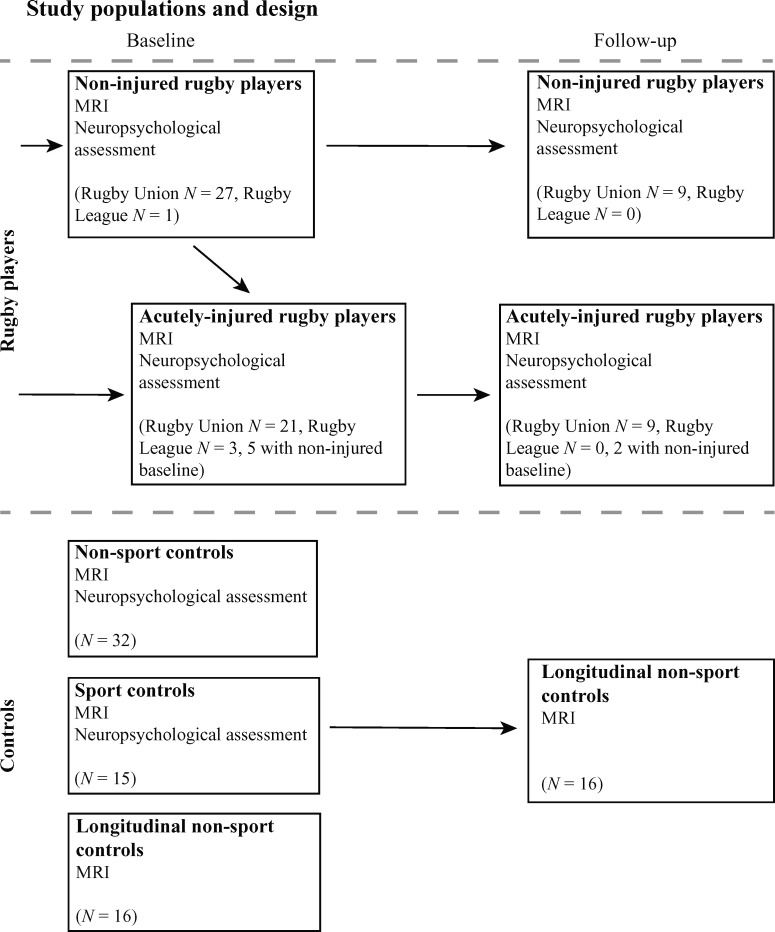
Overview of study cohorts. Thirty-seven elite male and three female rugby union players, and 4 male rugby league players were recruited from professional rugby clubs in England. Twenty-eight players had baseline study visits without an acute injury, while 21 players attended study visits shortly after a head. Post-injury study visits for acutely injured players were performed a maximum of 7 days after injury. Five players had both a baseline non-injury study visit and a study visit following an acute head injury, and 18 rugby players attended a second assessment. Three control groups were recruited to the study, including a Non-Sport and Sport Control group for cross-sectional comparison, and a separate group of Longitudinal Controls for longitudinal comparison. Longitudinal Controls only completed the structural imaging components of the study.

Thirty-seven elite male and three female rugby union players, and four male rugby league players were recruited from professional rugby clubs in the UK [mean age 25.2, standard deviation (SD) = 3.5, 41 males, 3 females]. Twenty-eight players had baseline study visits without an acute injury, while 21 players attended study visits shortly after a head injury as determined by medical professionals in line with Rugby Union and Rugby League guidelines. All head injuries met the Mayo Clinic classification of mild—probable TBI ^72^. Post-injury study visits for injured players were performed a maximum of 7 days after injury [mean days 4.7, SD = 1.2]. All study visits included an MRI assessment and battery of neuropsychological tests. Five players had both a baseline non-injury study visit and a study visit following a head injury (Fig. 1). Eighteen rugby players attended a second assessment a mean of 12.1 months later. Inclusion criteria were age 16–45, active rugby players with exclusion criteria comprising other major neurological or psychiatric issues (e.g. substance abuse, previous moderate-severe TBI) or contraindication to MRI.

Injury characteristics, including the total numbers of symptoms after injury and symptomatic days as well as the duration the player remained in the return to play protocol were recorded. Any head injuries in rugby union that met criteria for permanent immediate removal from play (e.g. loss of consciousness or ataxia as previously described^73^) were documented. Acutely injured players completed the SCAT-5 which includes a cognitive and neurological screen, and a symptom evaluation (symptom severity score out of 132). For all players, head injury history and length of professional career were collected from the professional sporting body, and where gaps existed, from players and the club. Self-reported alcohol intake was also recorded (Supplementary Table 1).

Healthy controls were screened according to the same exclusion criteria and were required to additionally have no self-reported history of mTBI. Three control groups were recruited to the study, including a Non-Sport and Sport Control group for cross-sectional comparison, and a separate group of Longitudinal Controls for longitudinal comparison. The Non-Sport Control group were a cohort of controls not playing sports at university or professional competition level. The Sport Controls group were a cohort of non-collision sport athletes recruited from university sport clubs. Inclusion criteria for the Sport Controls were age 16–45, active athletes at least 6 h of exercise per week with additional exclusion criteria of history of playing collision sports competitively. This included representing their high school competitively in rugby or soccer. Sport Controls comprised of 3 swimmers, 1 water polo player, 9 rowers, 1 cyclist and 1 weight lifter, with a median hours of exercise per week of 12 (IQR = 6.9). The Longitudinal Control group were the third cohort of controls and comprised of individuals who had participated in a longitudinal imaging study of outcomes after TBI which used aligned imaging and exclusion protocols.^65^ Longitudinal Controls only completed the structural imaging components of the study.

Recruitment was coordinated by local research facilities at the Hammersmith Hospital. All study participants provided written informed consent in accordance with the Declaration of Helsinki. Ethical approval was approved by the University College London research ethics committee (7385/001).

### Neuropsychological testing

Established paper neuropsychological tests sensitive to impairments after TBI^74^ were administered during study visits, including the Test of Premorbid Functioning (TOPF), Trail Making Test (TMT), Delis-Kaplan Executive Function System (D-KEFS Stroop), Hopkins Verbal Learning-Test (HVLT) and the Brief Visuospatial Memory Test-Revised (BVMT). Participants also completed a battery of cognitive tasks on an IPad as part of a subset of previously studied and described tasks.^75^^,^^76^ These included Visuospatial Working Memory, Paired Associates and Self-Ordered Search, Feature Match, Odd One Out and Spatial Planning. A visuospatial memory *Fractals* task on a touchscreen computer was also completed following a previously described protocol.^77^

### MRI acquisition

Participants were scanned with the following sequences: resolution structural T1-weighted MPRAGE with voxel dimensions of 1 mm^3^ (160 slices, matrix = 208 × 208, repetition time = 2300 ms, echo time = 2.98 ms, flip angle = 9°, FOV = 256 × 256 mm, GRAPPA = 2), T_2_ FLAIR (Fluid attenuated inversion recovery), T_2_ SWI (120 1.2-mm-thick transverse slices, TR = 28 ms, TE = 20 ms, FA = 15°, in-plane resolution = 0.8 × 0.6 mm, field of view = 225 × 225 mm) and DTI using a multishell 99 direction protocol (66 slices, isotropic 2 mm^3^ voxels, field of view = 25.6 cm × 25.6 cm, TR = 5000 ms, TE = 85 ms, b-value 1 = 700 s/mm^2^ with 30 directions, b-value 2 = 2000 s/mm^2^ with 60 directions, 6 direction b-value = 0 s/mm^2^ and 6 reverse phase encoding direction b-value = 0 s/mm^2^ volumes).

Longitudinal controls were scanned with the same structural sequences. Participants were all scanned on a Siemens 3T Verio (Siemens Healthcare) scanner using a 32-channel head coil. All scans were examined by a consultant neuroradiologist for the presence of focal lesions or microbleeds.

### Neuroimaging analysis

#### Diffusion data processing

**Figure 2:**
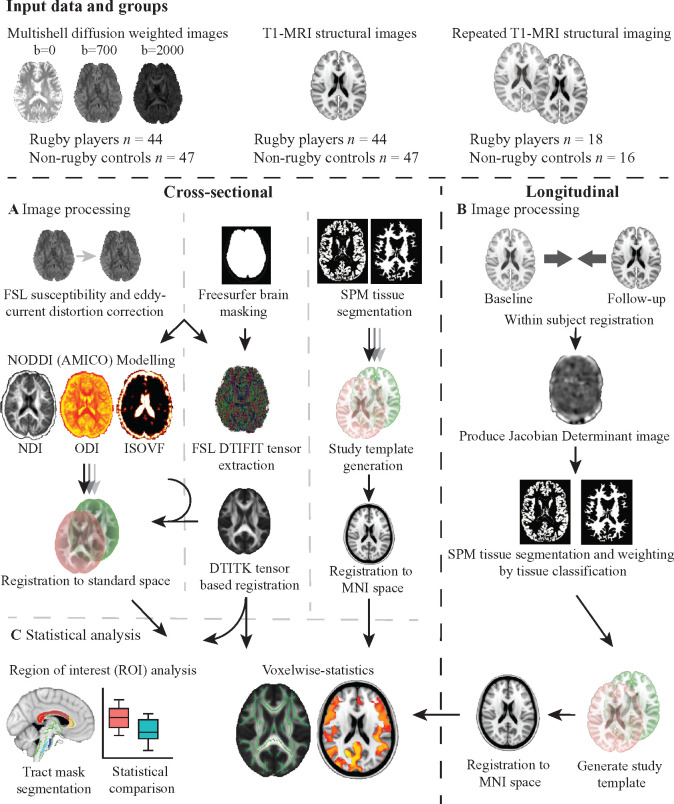
Overview of study methods. (**A**) Image processing of diffusion data followed the standard TBSS pipeline, including correcting for susceptibility and eddy current induced distortions and diffusion tensor fitting. Freesurfer was used to skull strip brains and remove non-brain voxels from the field map. Tensor-based registration was performed using DTI-TK involving the creation of a group template using affine and non-linear diffeomorphic registrations followed by registration of participant diffusion imaging to the template. Images were all warped to 1 mm isotropic space and the mean fractional anisotropy map produced was thresholded at 0.2 to produce a white matter skeleton. Subject fractional anisotropy and mean diffusivity data were projected onto the skeleton and tract level data generated using the Johns Hopkins University white matter atlas. Neurite density index (NDI), orientation dispersion index (ODI) and isotropic volume fraction (ISOVF) maps were generated using NODDI modelling using the Accelerated Microstructure Imaging via Convex Optimization (AMICO) framework. NDI, ODI and ISOVF maps were registered to DTI-TK-space using the transformation matrix generated during the DTI-TK pipeline. Images were visually inspected at the brain extraction, eddy current correction and tensor registration stage for quality control checks. Image processing of cross-sectional structural data used SPM to segment T1 images into tissue probability maps. A study-specific template was generated using DARTEL registration for non-linear spatial normalization and then affine registered to MNI152 space. All images were then normalized to MNI152 space via the study template. (**B**) Processing of longitudinal structural images involved within-subject registration of baseline and follow-up images and generation of a within-subject temporal average image and Jacobian determinant image. The Jacobian determinant image represented a voxelwise statistic of spatial expansion and contraction required to match baseline and follow-up images. Average images were then segmented into grey and white matter and Jacobian determinant images normalized by tissue probability. A study-specific longitudinal template was generated with DARTEL and affine registered to MNI152 space. Individual average images were then normalized to MNI152 space via the longitudinal study template. (**C**) Statistical analysis was carried out in both a region of interest and voxelwise manner. Region of interest analysis involved sampling of mean diffusion and structural metrics of interest within pre-defined regions of interest in spatially standardized images. Voxelwise analysis was carried out using Randomise (FSL) with 5000 permutations using the threshold-free cluster enhancement and adjusting for age, gender and intracranial volume (cross-sectional structural only).

Diffusion weighted images were processed following the standard TBSS pipeline in the FMRIB Software Library (version 5.0.8),^78^ including correcting susceptibility and eddy current induced distortions^79^ and diffusion tensor fitting (Fig. 2). Freesurfer^80^ was used to skull strip brains and remove non-brain voxels from the field map. Tensor-based registration was performed using DTI-TK^81^ involving the creation of a group template using affine and non-linear diffeomorphic registrations followed by registration of participant diffusion imaging to the template. Images were warped to 1 mm isotropic space and the mean FA map produced was thresholded at 0.2 to produce a white matter skeleton. Subject FA and mean diffusivity (MD) data were projected onto the mean FA skeleton and tract level data generated using the Johns Hopkins University (JHU) white matter atlas. Neurite density index (NDI), orientation dispersion index (ODI) and isotropic volume fraction (ISOVF) maps were generated using NODDI modelling using the Accelerated Microstructure Imaging via Convex Optimization (AMICO) framework.^82^ NDI, ODI and ISOVF maps were registered to DTI-TK-space using the transformation matrix generated during the DTI-TK pipeline. Images were visually inspected at the brain extraction, eddy current correction and tensor registration stage. Subjects with poor quality data or un-correctable artefacts were removed.

#### Subject-level diffusion analysis

Mean FA was calculated for selected large white matter tracts based on tract size, reproducibility and sensitivity.^83^ The mean and standard deviation of FA for each tract region of interest (ROI) across the Non-Sport Control cohort was then used to calculate subject *Z*-scores for each ROI. *Z*-scores were converted to *P*-values (two-tailed, with 95% confidence interval) and corrected for multiple comparisons across all tract ROIs using FDR. Abnormalities in subjects’ FA were determined by tracts that have significantly lower or higher FA in the subject compared to the control group, *P* < 0.05, FDR corrected. Subject-level and ROI analysis was only conducted for FA, and not other diffusion metrics.

#### Voxelwise diffusion analysis

Voxelwise analysis of skeletonised diffusion metrics was conducted using the general linear model with non-parametric permutation testing (5000) in FSL Randomise.^84^ Diffusion weighted voxelwise results reported are corrected for multiple comparisons at a threshold of *P* < 0.05 with age and gender included as a nuisance covariate.

#### Cross-sectional structural data processing and analysis

Volumetric data were analysed using standard approaches^65^ (Fig. 2). SPM 12 (UCL) was used to segment T1 images into grey matter, white matter and CSF, and to calculate volumes of these tissue classes at baseline and follow up. Cross-sectional analysis involved the generation of a study template using SPM DARTEL non-linear registration^85^ before affine registration of segmented images to MNI152 space, with normalization of volume and smoothing using an 8 mm kernel. Tract level data were generated using eroded JHU white matter atlas’ masks to sample tissue volumes. Cross-sectional voxelwise analyses were conducted using non-parametric permutation testing (5000) with age, gender and intracranial volume included as nuisance covariates.

#### Longitudinal structural data processing and analysis

Longitudinal volumetric analysis was performed using the SPM12 longitudinal registration tool. Baseline and follow-up T1 images were iteratively co-registered to produce a temporal average image, with the transformation required to move from the baseline to follow-up image encoded in the JD of the expansion/contraction at a voxel-wise level.^62^ JD was then weighted by the interval between baseline and follow-up to give an annualized rate of change and further weighted by tissue class. A study-specific template was generated using SPM DARTEL’s non-linear registration and tissue-specific JD images normalized to group space before affine registration to MNI152 space with normalization of volume and smoothing using an 8 mm kernel. Tract level data were generated using skeletonised JHU white matter atlas’ masks in MNI152 space. Voxelwise analysis was conducted using non-parametric permutation testing (5000) with age included as a nuisance covariate and results reported are corrected for multiple comparisons at a threshold of *P* < 0.1. Voxelwise correlation between JD and DTI metrics were conducted after registration of DTI images to MNI152 space, using non-parametric permutation testing (5000).

### Statistical analysis

Whole brain summary and voxelwise measures were calculated by adding grey and white matter tissue data. Summary measures of brain volume were normalized for participant head-size by dividing them into total intracranial volume (grey matter + white matter + CSF). Statistical tests were completed in the open-source software package R ^86^. Planned analyses included the comparison of demographics, neuropsychological performance and diffusion metrics in rugby players and all controls as a group, and sub-analysis between sub-acutely injured players and non-acutely injured players (Fig. 1). An individualized assessment of FA was planned to identify abnormalities in DTI using methods previously described ^83^. An analysis of brain atrophy comparing rugby players and a sub-group of controls with longitudinal data were also planned using previously described methods.^65^

Non-sport and sport control groups were combined as an ‘All controls’ group for comparisons with the largest *N.* This was done for a comparison of demographics, neuropsychological test performance and voxelwise diffusion and cross-sectional volumetric imaging analyses. Further sub-analyses were performed using the Sport and Non-Sport Control groups individually to investigate the effect of sports participation and test if the group-wise differences in diffusion measures remained. Rugby players were also split into sub-acutely and non-acutely injured players to investigate the effects of a sub-acute injury on neuropsychological performance and imaging metrics. Longitudinal controls, who were not screened for sports participation, were used for longitudinal volumetric imaging analyses comparisons against a group of both sub-acutely and non-acutely injury players, combined to maintain large enough group sizes for powering. A subgroup analysis was performed using only male Rugby Union players and utilised the same methodology as the main analyses but included only male non-sport and sport controls for cross-sectional comparisons.

Group differences between all rugby players and all controls in demographics and neuropsychological performances were investigated using *t*-tests, with effect sizes reported using Cohen’s d. The effect of sub-acute injury and sports participation on neuropsychological test performance was assessed using an analysis of variance with Tukey multiple *post* *hoc* comparisons of means. FDR correction for multiple comparisons was used. Details on neuroimaging statistical analysis are detailed in the neuroimaging section. Pearson’s correlation was used to test the relationship between JD and FA, and between the neuropsychological test score performances and neuroimaging metrics. Categorical variables were compared using the Chi-squared test and effect sizes reported using Cramer’s V. In cases where the normality assumption was violated, the Wilcoxon test was used. The data were not manipulated to account for missing data and design and analysis not prespecified publically.

### Data availability

The data that support the findings of this study are available from the corresponding author, upon reasonable request.

## Results

### Subjects

From 26 July 2017 until 1 March 2019, 44 elite level rugby players were recruited into the study. Twenty-one players were assessed shortly after a mild TBI that was sustained whilst playing professional rugby. On average these players were 4.7 days post-injury. Sub-acutely injured players presented with a mean symptom severity score of 10.3 on the SCAT-5 during their pitch side assessment at the time of injury and had an average return to play duration of 7.2 days (Supplementary Table 1). Demographic and physical characteristics were well matched between sub-acutely injured and non-acutely injured rugby players. Eighteen rugby players attended a second assessment a mean of 12.1 months later and players who returned for a follow-up were not significantly different in demographics, baseline neuropsychological test performance or any imaging measures compared to players who did not return for follow-up.

Rugby players were compared to three control groups. A non-collision sport cross-sectional (Sport Controls), a non-sport cross-sectional (Non-Sport Controls) and non-sport longitudinal control group (Longitudinal Controls). Sport Controls were younger than rugby players (*t* = −3.44, *P* = 0.0022, *d* = 1.05) with no difference in proportion of males to females. Non-Sport Controls were well matched for age, but had a lower proportion of males to female (*X*^2^= 10.7, *P* = 0.001, *V* = 0.42) than the rugby players. Longitudinal Controls were older than the rugby players who returned for their longitudinal assessment (*t* = 6.28, *P* < 0.001, *d* = 2.16) but were matched for gender. Alcohol intake was not significantly different between rugby players and controls.

### Rugby players showed lower neuropsychological test performance at baseline

**Figure 3:**
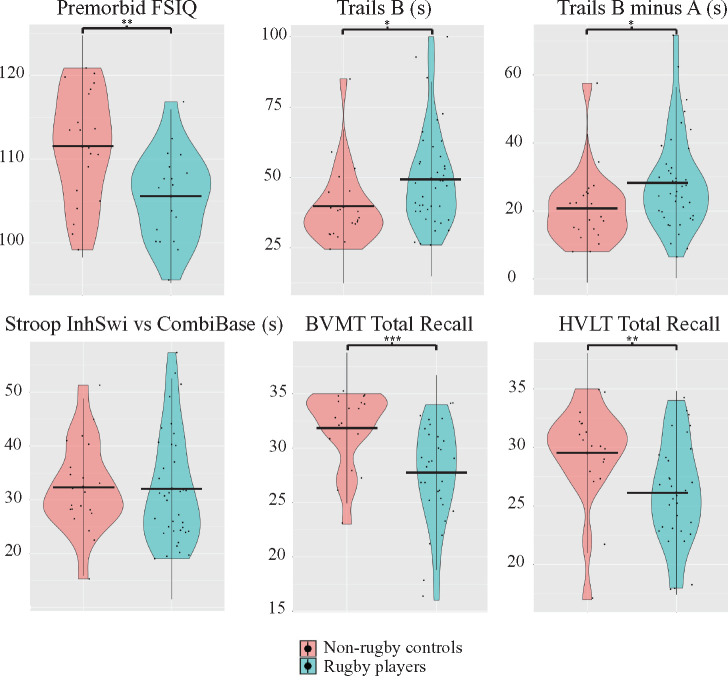
Neuropsychological performance in all controls and rugby players. Grouped violin plots of rugby player and non-rugby control performance in select neuropsychological tests. The width of the coloured area represents the distribution of scores, with the mean defined by the black horizontal lines. Independent samples *t*-tests were conducted, with * = uncorrected *P *<* *0.05, ** = uncorrected *P *<* *0.01, *** = uncorrected *P *<* *0.001. Premorbid FSIQ is a composite score of intelligence, Trails B and Trails B minus A are tests of speed of processing, Stroop InhSwi versus CombiBase is a composite score indicative of executive function, BVMT Total Recall and HVLT Total Recall are scores of immediate memory. BVMT, Brief Visuospatial Memory Test; CombiBase, Combined Baseline; FSIQ, Full Scale Intelligence Quotient; HVLT, Hopkins Verbal Learning Test; InhSwi, Inhibition Switch.

Rugby players as a group showed lower visual memory immediate recall (*t* = 3.64, *P* = 0.0083, *d* = 0.99) and lower premorbid intelligence scores (FSIQ; *t* = 3.2, *P* = 0.030, *d* = 0.99) compared to Non-Sport and Sport controls as a single group (Non-Rugby controls) (Fig. 3). Rugby players also showed trends towards slower performance on two tests of processing speed (Trails B, Trails B minus A) and poorer performance on verbal memory immediate recall, however, these did not survive correction for multiple comparisons. There were no differences between players and controls in computerized testing performance or on measures of executive function.

### Neuropsychological test performance in rugby players was not influenced by sub-acute injury

To assess if these differences were present in both sub-acutely injured players and non-acutely injured players, and if there was an effect of sport participation in controls, we compared baseline performance between the 4 groups. A significant effect of group was found for premorbid intelligence scores (FSIQ; *F*(3,37) = 5.74, *P* = 0.03). *Post hoc* tests showed that these effects were driven by lower FSIQ scores in both sub-acutely injured rugby players (*P* = 0.0035) and non-acutely injured players (*P* = 0.0095) when compared to Non-Sport Controls. There were trends towards effects of group on visual memory immediate recall and verbal memory immediate recall, however, these did not survive correction for multiple comparisons. There were no differences between neuropsychological test scores of non-acutely and sub-acutely injured rugby players at baseline, nor any differences between baseline and follow-up scores within players who returned for a second visit.

### Abnormal white matter integrity in rugby players

**Figure 4:**
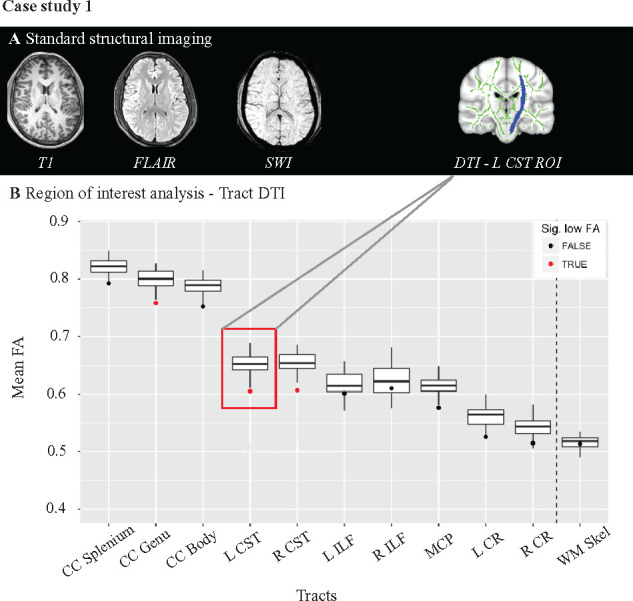
Subject level analysis of fractional anisotropy: Case study 1. (**A**) Routine MRI scans including T1-MPRAGE (magnetization-prepared rapid acquisition with gradient echo), SWI (susceptibility weighted imaging) and FLAIR (fluid attenuated inversion recovery) of the player showed no abnormalities. (**B**) An individualized approach to DTI analysis. Mean FAs from specific white matter tracts are plotted; highlighted in red and inset above are the areas sampled corresponding to the left corticospinal tract. The boxplots represent the values found using the Non-Sport Control cohort (*n* = 32). Single points on the plot represent the individuals mean FA value within the specific tract. Black dots represent where a value is not abnormal, while red points indicate where an individual has a significantly abnormal FA compared to the control cohort. Abnormal FA is defined by using a *z*-scoring approach, comparing the individual to the Non-Sport Control cohort, *P* < 0.05, 95% confidence interval, FDR corrected. CC, corpus callosum; CR, corona radiata; CST, corticospinal tract; ILF, inferior longitudinal fasiculus; MCP, middle cerebellar peduncle; WM Skel, whole white matter skeleton.

White matter structure was assessed using diffusion, volumetric and susceptibility weighted MRI scans. Three baseline DTI scans of rugby players and 2 Sport Control DTI scans were discarded after quality control checks. We investigated the white matter structure at the individual level by calculating the mean FA from a number of large white matter tract and the whole white matter skeleton and comparing results to control ranges. This approach is illustrated in the case study presented in Fig. 4. Conventional MRI of this rugby player with a sub-acute injury showed no evidence of abnormalities. In contrast, FA averaged across specific white matter tracts showed abnormalities in the genu of the corpus callosum, and left and right corticospinal tract, with low FA characteristic of white matter abnormality.

**Figure 5:**
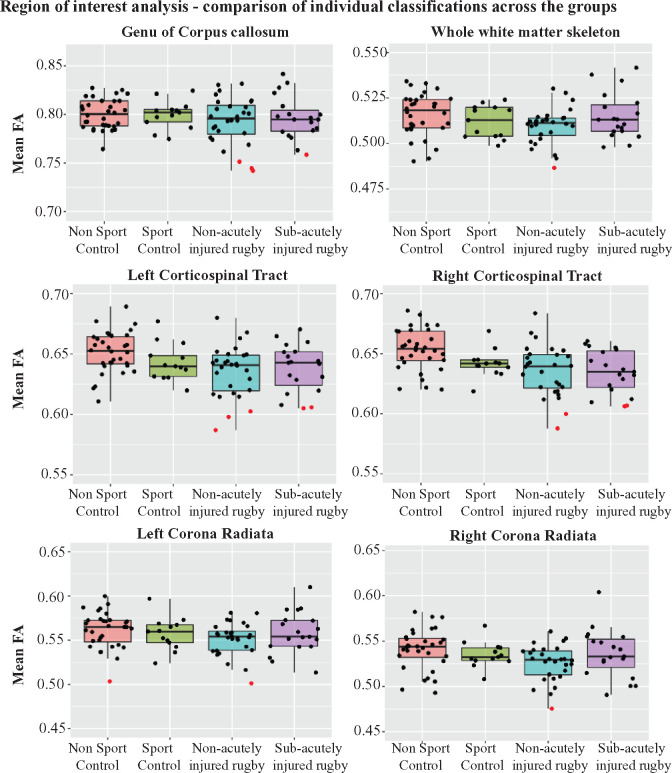
Results of individualized analysis of fractional anisotropy across the groups. Mean FAs from select white matter tracts where abnormalities were observed are plotted. Single points on the plot represent an individuals’ mean FA value within the specific tract. Black dots represent where a value is not abnormal, while red points indicate where an individual has a significantly abnormal FA compared to the Non-Sport Control cohort. Individuals with at least one abnormal value across the tracts are determined to be DTI abnormal. Across the groups, a higher proportion of rugby players with abnormalities compared to controls can be observed.

Across the whole cohort, there was a higher proportion of rugby players with DTI abnormalities defined in this way than controls (*X*^2^ = 3.97, *P* = 0.046, *V* = 0.21). Seventeen per cent of rugby players (7/42) had abnormalities in mean FA in at least one of the sampled tracts, compared to 3% of Non-Sport Controls (1/32). Sport Controls showed no abnormality in FA (Fig. 5). The prevalence of abnormalities was highest in the corpus callosum (3/7, 43%) and the corticospinal tracts (5/7, 71%). There were no differences in alcohol intake between players with diffusion abnormalities and those without.

**Figure 6:**
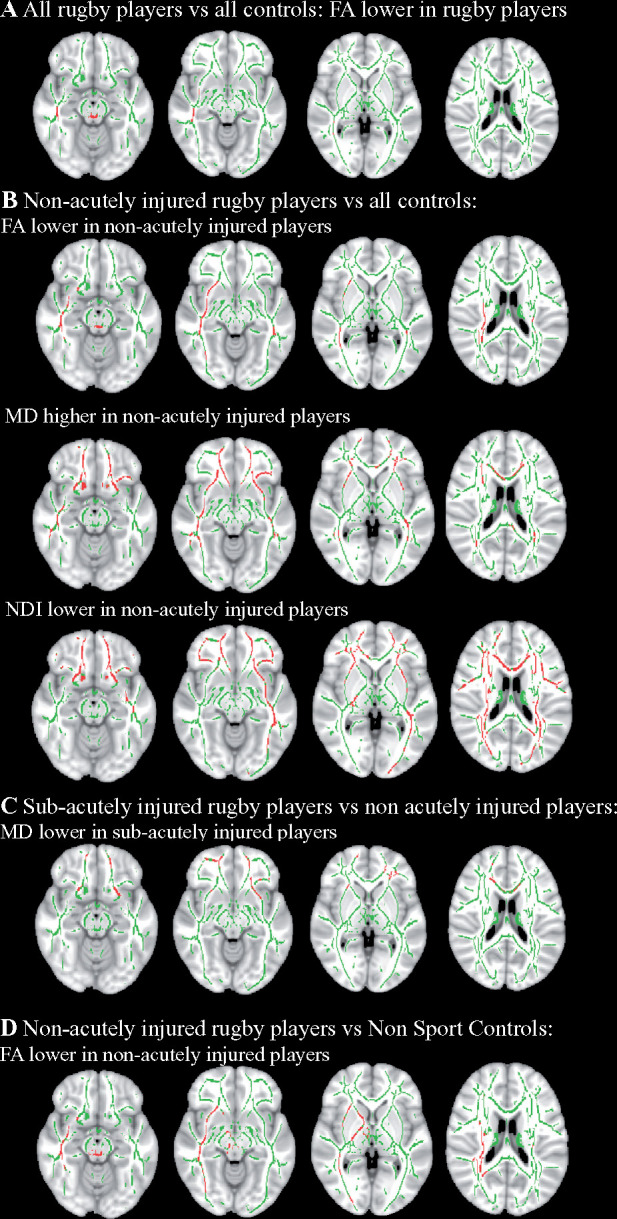
Voxelwise comparison of diffusion metrics between groups. Voxelwise comparisons were conducted using FSL Randomise with age and gender added as covariates. Green denotes the white matter skeleton and areas in red indicate a result where corrected *P *<* *0.05. (**A**) Comparisons of fractional anisotropy (FA) between all rugby players (*n *=* *42) and all controls (*n* = 45) showed regions of lower FA in rugby players compared to controls. No differences were seen in other diffusion metrics. (**B**) Regions of lower FA, higher mean diffusivity (MD), and lower neurite density index (NDI) were observed in non-acutely injured rugby players (*n *=* *27) when compared to all controls. (**C**) Elevated FA was observed in sub-acutely injured players (*n *=* *19) when compared to non-acutely injured players (*n *=* *24) excluding multiple observations of the same player. No differences were seen in other DTI metrics. (**D**) When splitting the control group, non-acutely injured rugby players still showed areas of reduced FA relative to Non-Sport Controls (*n *=* *32). No differences were observed in other DTI metrics, nor between Non-Sport Controls and sub-acutely injured players or between Sport Controls (*n *=* *13) and either rugby group.

Voxelwise group comparisons of diffusion MRI also provided evidence for altered white matter integrity in rugby players. Voxelwise cross-sectional comparison of all rugby players to all controls (Non-Sport and Sport Controls combined) showed reductions in FA in the posterior thalamic radiation, left posterior corona radiata, left posterior limb of internal capsule and the superior cerebellar peduncle bilaterally (Fig. 6A). No significant differences were observed for the other DTI metrics investigated (MD, NDI, ODI and ISOVF).

### Differences in white matter integrity between non-acutely and sub-acutely injured players

To disentangle the effects of a sub-acute injury, we investigated diffusion changes in sub-acute and non-acutely injured players separately. Out of the 7 players with DTI abnormalities, 5 were non-acutely injured and 2 were scanned sub-acutely after injury. Voxelwise analysis showed that imaging abnormalities were most prominent in non-acutely injured players. Compared to all controls, reductions in FA in the non-acutely injured group were seen in the corona radiata and sagittal stratum, as well as elevations of MD in the genu of the corpus callosum, corona radiata, posterior thalamic radiation, retrolenticular part of internal capsule and sagittal stratum. Furthermore, lower NDI was seen in widespread white matter regions in the non-acutely injured players (Fig. 6B). In contrast, sub-acutely-injured rugby players showed no difference in diffusion metrics when compared to all controls.

We then compared the sub-acutely injured players with non-acutely injured rugby players. MD was reduced in sub-acutely-injured players compared to non-acutely injured players within the genu of the corpus callosum, retrolenticular part of the internal capsule, corona radiata and external capsule (Fig. 6C). There were no differences in the other DTI metrics.

We split the control groups to investigate the effect of sporting activity on diffusion measures. There were no differences between observed in any of the DTI metrics between the Non-Sport Controls and Sport Controls. We investigated if the diffusion differences were still present using only Sport or Non-Sport Controls. Reductions in FA were still observed in non-acutely injured rugby players when compared to only Non-Sport Controls in the corona radiata, posterior thalamic radiation, sagittal stratum and corticospinal tract (Fig. 6D). There were no reductions in other DTI metrics, differences between sub-acutely injured players and Non-Sport Controls nor differences when comparing the Sports Control group with either rugby group.

### Microbleed evidence for DVI

**Figure 7:**
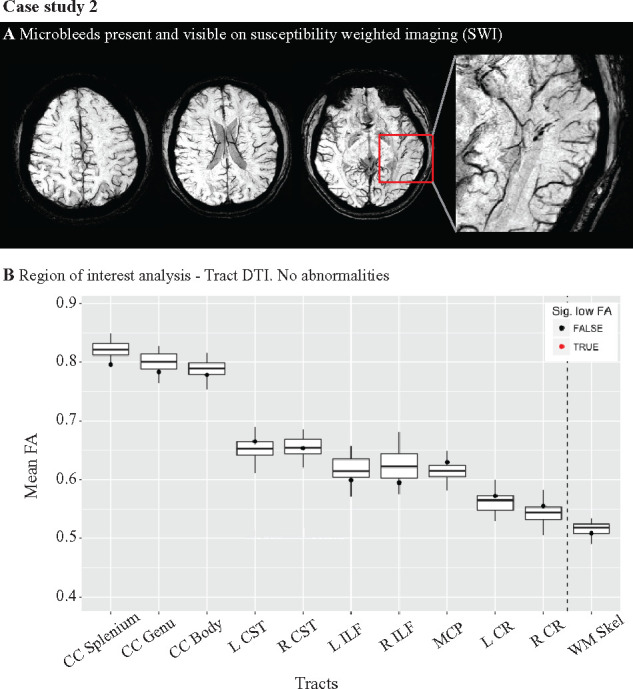
Presence of microbleeds on susceptibility weighted imaging: Case study 2. (**A**) A player with a sub-acute injury presented with no abnormalities on routine structural MRI scans, but had evidence of microbleeds (highlighted in the magnified imaged) on susceptibility weighted imaging (SWI). (**B**) Utilising the individualized approach to DTI analysis, no abnormalities were found in FA for this player. CC, corpus callosum; CR, corona radiata; CST, corticospinal tract; ILF, inferior longitudinal fasiculus; MCP, middle cerebellar peduncle; WM Skel, whole white matter skeleton.

Three rugby players had microbleeds, 2 of whom were sub-acutely injured, while none were present in Non-Sport Controls (Fig. 7). One Sport Control showed microbleeds. FA abnormalities were not observed in the players with microbleeds. Hence, 23% of players (10/44) had evidence of diffuse axonal or DVI. Longitudinal SWI was stable in individuals with follow-up scans, with no change in microbleed incidence or evolution of microbleeds on SWI scans.

### Rugby players show abnormal white matter volume change over time

We assessed volumetric differences between the rugby players and controls. Cross-sectional comparisons were made between the groups at baseline. A voxelwise analysis of grey and white matter volumes showed no significant differences at baseline between players and all controls. There were no differences between the control groups, or between non-acutely injured and sub-acutely injured rugby players.

**Figure 8:**
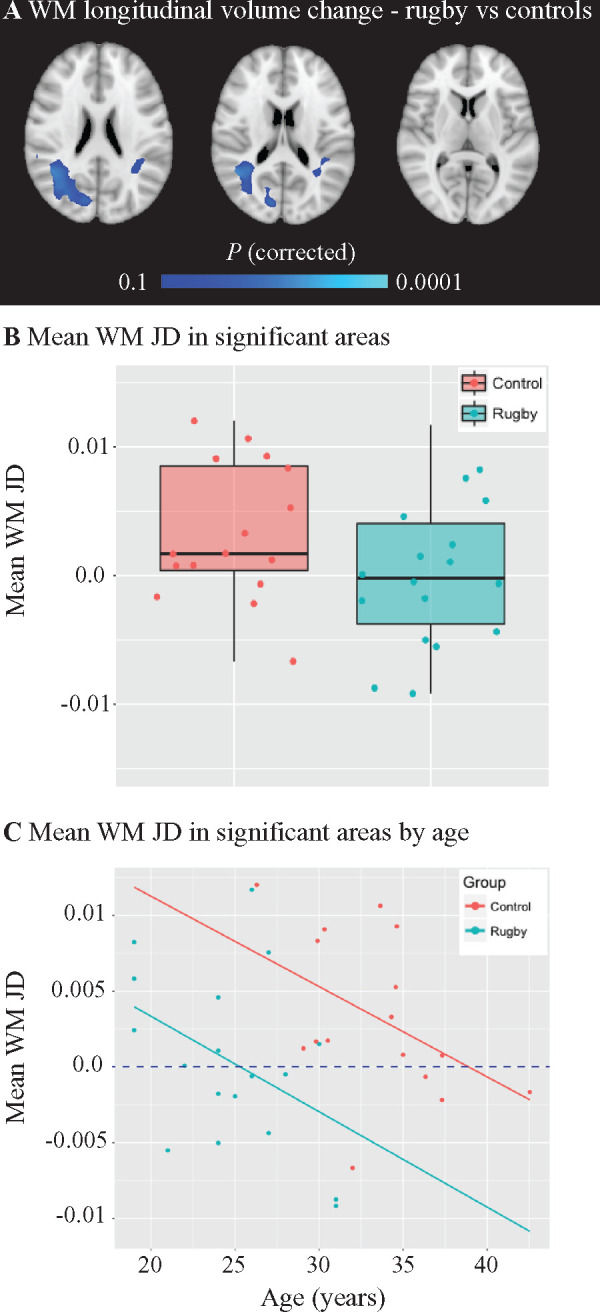
Longitudinal changes in volume in rugby players. Voxelwise comparisons were conducted using FSL Randomise with age as a covariate and results shown for areas where corrected *P *<* *0.1 (one tailed *t*-test). (**A**) Lower annualized Jacobian Determinant (JD) in rugby players (*n = *18) compared to Longitudinal Controls (*n *=* *16) was observed in the white matter. (**B**) When plotting the JD values within the significant areas, values between rugby players (blue) and Longitudinal Controls (red) look similar. (**C**) However, when plotting against age, a proportion of rugby players have JD values that appear lower than what would be expected for individuals of that age, suggesting there are altered trajectories for white matter volume change in rugby players.

We then investigated whether there was any evidence for abnormal brain volume changes in rugby players using a sensitive measure of brain volume change over time (Jacobian Determinant, ‘JD’). Voxelwise comparison of JD maps between rugby players who attended 2 visits and the Longitudinal Control group showed lower white matter JD in rugby players, specifically within the lateral occipital cortex and post central gyri (Fig. 8A). A lower JD suggests that either the rate of volume expansion was lower in rugby players than controls, or players were losing volume at a higher rate than controls. Longitudinal Controls were slightly older than the rugby players (mean age in years 33.3 versus 24.8, *t* = 6.28, *P* < 0.001), and so would be expected to have lower JD values than the rugby players. However, the reverse was true. Sampling the mean JD values from these regions revealed that 50% of players (9/18) had mean JD values below zero (indicating brain volume loss) compared to 25% of controls (4/16) (Fig. 8B). These results suggest that the rugby players had an abnormal trajectory of white matter volume change for individuals of that age (Fig. 8C). Mean JD within the grey, white matter or whole brain did not correlate significantly with head injury history.

### White matter integrity and longitudinal volume change in rugby players

We tested whether areas of the white matter showing evidence of abnormal volume changes over time (low JD) in rugby players were associated with abnormalities in white matter integrity (low FA). We sampled the diffusion metrics and JD values within the white matter tracts in areas where there was a significant difference in JD between players and controls and found no correlation between baseline FA, MD or NDI with JD in these areas. Additionally, there were no significant correlations when restricting analysis only to non-acutely injured rugby players.

### White matter volume changes, white matter integrity and neuropsychological performance

We also explored the relationship between atrophy rates, white matter integrity and cognitive function. Investigating tasks which showed differences at baseline, no correlations were found between the mean FA, MD or NDI in white matter tract ROI’s and neuropsychological test performance. Rugby players who showed abnormalities in at least one white matter tract ROIs (*n *=* *7) did not perform significantly differently in neuropsychological tests, including premorbid intelligence scores, compared to players not classified as abnormal on their individualized DTI. No correlations were found either with mean JD in grey, white matter or whole brain and neuropsychological test scores. We investigated if premorbid intelligence influenced neuroimaging parameters. We removed 3 non-sport controls at the higher range of FSIQ scores so that the non-sport controls (*n* = 29) and rugby players (*n* = 42) were matched for premorbid intelligence. Running the individualized DTI assessment with FSIQ matched controls, players previously identified as abnormal on DTI remained abnormal. Furthermore, FSIQ scores were not correlated with mean FA within rugby players or controls.

### Subgroup imaging analysis in male Rugby Union players only

We investigated if there were any differences in the results when looking at scans from male rugby union players only (*n *=* *37) and included only male controls for the cross-sectional comparison. A similar pattern of abnormalities was observed, with 11% of rugby players (4/35) with DTI abnormalities and 8% with microbleeds (3/37). In total, 19% of players (7/37) had evidence of diffuse axonal or DVI. Voxelwise comparisons of diffusion metrics showed similar results, with reductions in FA and NDI in non-acutely injured rugby players persisting. With 3 players excluded from the longitudinal volumetric analysis (*n *=* *15), the area of abnormality in JD within the white matter remained but was slightly reduced in size.

## Discussion

Our longitudinal observational study shows abnormalities of brain structure associated with elite rugby participation. DTI and SWI were used to assess white matter structure and evidence of axonal injury was seen in several players studied. Across the group of rugby players studied abnormalities of FA and other diffusion measures were seen. In contrast, sport playing controls were not classified as showing abnormalities of FA. We also studied changes in brain structure over time. Abnormal trajectories of white matter volume were seen in elite rugby players, suggesting an impact of elite rugby participation on brain development. To our knowledge, this is the first study to investigate MR imaging changes in active elite rugby players.

Diffusion imaging abnormalities of FA, neurite density and MD were seen in the rugby group. Similar directions of change are seen in other mild TBI patients scanned in the chronic stage^40^^,^^45^^,^^87^ and longitudinally^88^ after an injury. We used an individualized approach to DTI analysis to identify players with neuroimaging abnormalities.^83^ This allowed us to investigate the structure of a number of large white matter tracts in a focussed way. Twenty-three per cent of the rugby players showed signs of either diffuse axonal or DVI. The majority of the players with evidence of DAI showed abnormalities within the corticospinal tract. Reductions in FA are associated with abnormalities of motor function in other contexts.^89^^,^^90^ Although this was not explicitly assessed, the players studied did not show any evident abnormalities of motor function and all players were successfully competing at an elite level. However, retired rugby players have been shown to display reduced fine motor control ability.^18^^,^^91^ Therefore, it is possible that reductions of FA within the corticospinal tract might be significant in terms of future motor function.

Abnormalities of white matter structure are correlated with clinical features of injury severity and neuropsychological test performance following moderate-severe TBI, with evidence of DAI (low FA) usually associated with cognitive impairment.^24^^,^^34^^,^^65^ Although the rugby players we studied showed did show poorer performance on a number of cognitive tests, cognitive performance did not correlate with white matter abnormalities. Players with white matter abnormalities did not perform significantly worse than those classified as normal. Rather than effect of a rugby participation or sub-acute injury, the difference in performance observed across a select number of neuropsychological tests may have been related to group differences in IQ and appears unrelated to injury or imaging abnormalities within this study.

Multi-shell diffusion-MRI was acquired. This allowed Neurite Orientation Dispersion and Density Imaging (NODDI) to be performed. NODDI is an advanced diffusion MRI technique was used to calculate a range of diffusion metrics that potentially provide more sensitive and specific diffusion metrics of white matter pathology. NODDI separates the diffusion signal from three different tissue compartments: intra-neurite (restricted diffusion), extra-neurite (hindered diffusion) and cerebrospinal fluid (free diffusion) to extract a NDI, ODI and ISOVF.^92^ Sub-acutely after a mild TBI, studies have observed reduced NDI,^93^ which has correlated with reductions in FA and increased symptom load.^42^ In athletes not acutely injured, but with a history of injury, studies have found abnormalities in FA which were negatively correlated to ODI.^40^^,^^94^ We extend these findings by showing widespread reductions in NDI in our non-acutely injured rugby players, but not in the sub-acutely injured players, with no differences in ODI or ISOVF. These results are suggestive of lower white matter tissue density and that the FA and MD findings are not primarily driven by variations in free water volume.

Moderate-severe TBI produces a progressive loss of brain volume, particularly affecting the white matter.^65^^,^^95^ This is a marker of the progressive neurodegenerative process that can be triggered by TBI.^96^ Abnormal white matter volume changes were observed in the rugby group. Reductions in a region of white matter volume, as defined by a negative JD, were seen in around 50% of the rugby players studied, and a higher rate of white matter volume loss was seen across the group when compared to a control group who were slightly older. White matter volume is expected to increase until the age of around 40,^64^^,^^97^ and this pattern was seen in our control group who had longitudinal imaging. The rugby players studied had an average age of 25 years old and the oldest was 31 years old. In this age range reductions of white matter volume are abnormal and may be an early sign of an active neurodegenerative process that might increase the risk of neurodegenerative disease in later life. The pathological correlates of abnormal white matter volume we detected with advanced neuroimaging in rugby players is unknown and requires more investigation. However, evidence of CTE in retired professional rugby players has been previously observed,^21–23^ and in deceased professional Scottish football players where head injury risk is lower but exposure to heading the football is seen as a unique sport-specific risk factor, a five-fold increased risk of developing Alzheimer’s disease was identified.^61^

The current focus of head injury management programmes in sport is the identification of defined observable signs, such as loss of consciousness and ataxia,^98^ and assessment for the presence of characteristic symptoms and alterations from baseline in cognitive function and balance. Effective surveillance at the elite and community levels of Rugby Union is overseen in the UK by the Rugby Football Union and any signs or symptoms of mTBI routinely lead to a player’s removal from play and a graduated return to play once these have resolved. Animal studies show that early markers of neurodegeneration such as tau pathology, vascular damage and neuroinflammation can be produced by mild injuries of the type frequently experienced by professional rugby players.^7^^,^^99–102^ However, an important observation is that this pathology can be seen in the absence of concussive symptoms. Players in a range of sports are repeatedly exposed to head impacts in matches and training that may in themselves be sufficient to result in the subsequent development of neurodegenerative pathology. However, these impacts would not trigger removal from play and are not routinely measured over a player’s career. Further studies of both active and retired rugby players are needed to investigate whether these subthreshold impacts as well as reported mTBIs are associated with evidence of brain injury and to ascertain how cumulative injury burden relates to neurodegeneration and ongoing brain health.

We compared rugby players with a recent history of mTBI to those without. Reduced MD was seen in sub-acutely injured rugby players compared to non-acutely injured players, although there were no differences when compared to the control group. This is in keeping with several previous studies of sport mTBI, which have shown reduced diffusivity alongside elevated FA in recently injured players.^42^^,^^103^^,^^104^ Other diffusion metrics did not show any abnormalities sub-acutely after injury. Previous studies have described the dynamic nature of diffusion changes seen in the early stages of TBI where FA increases initially in the acute to sub-acute period.^27^ However, sub-acutely injured players did not show abnormalities of FA compared to controls. Similar findings have been described in other studies of non-acutely injured contact sport athletes.^105–107^ In the context of reduced FA observed in our non-acutely injured players, the absence of evidence for diffusion changes in sub-acutely injured players may be due to the acute effects being occluded by longer term reductions in FA.

Three players also had microbleeds visible on their SWI, an abnormality not seen in any of our non-sporting controls, but observed in 1 sporting control. Microbleeds are a marker of DVI and are produced by small perivascular haemorrhage that can be missed by conventional imaging and that remains visible on SWI because of the persistence of haemosiderin laden macrophages in the perivsascular region.^52^^,^^108^ Previous studies of have reported the presence of microbleeds after mild TBI, including in contact sport athletes.^56^^,^^109^ We extend these findings by showing that microbleeds can occur in the absence of diffusion abnormalities and vice versa. The distinct information identified by these two imaging methods has recently been shown in moderate-severe TBI.^52^ This illustrates that DVI and DAI can occur independently after TBI and that SWI and diffusion MRI provide complementary information about the presence of subtle brain injury.

There are some limitations to our study. Our longitudinal control group were non-sport controls, meaning that the effect of exercise was not controlled for in our longitudinal analyses. However, we do not believe this likely to have a large effect on the comparison as evidence suggests exercise should lead to higher grey and white matter volume.^110–112^ A further limitation to our study was that we had gender and age differences in our control groups when compared to the rugby players. We minimised this effect by modelling age in all groupwise analyses, and gender where appropriate. One potential limitation is that diffusion metrics such as FA have been shown to be related to premorbid intelligence scores,^113^^,^^114^ which were higher in our control group. This did not appear to be a major confound for our results, as a sensitivity analysis showed no difference in classification of DTI abnormalities in rugby players when the groups were matched for FSIQ, and intelligence scores were not correlated to FA. Our non-collision sport control group were also not professional athletes and not matched for BMI which limits our ability to exclude the effect of high-level sporting participation on our results. We only had a small sample of players who had a followed-up visit, meaning we were not well powered to investigate the effect of a sub-acute injury on longitudinal volume change, which may be influenced by the resolution of oedema or inflammation, and lacked longitudinal DTI to help interpret the volumetric changes. Our sports control group was small and may have been underpowered to detect differences in a group wise analysis. We therefore utilised an individualized analysis to detect abnormalities in FA, and did not observe any abnormalities in our sports control group compared to 17% of rugby players.

It is important to note that our results in adult professional rugby union and league players are not directly comparable to play at the community or youth levels. The overall health benefit of participating in sports and physical exercise have been well established including the reduction in mortality and chronic diseases such as dementia,^115^^,^^116^ and have been explored in detail within rugby.^117^ In the general professional sport setting, evidence of long-term health benefits includes lower all-cause mortality.^61^^,^^118^ The clinical implications on an individual level of the imaging changes associated with elite rugby participation are unclear. In retired rugby players, a lower risk of cardiovascular disease, with no evidence of exposure to repetitive head injuries nor participation in elite rugby affecting later life mental health, social or work functioning was found.^18^

In conclusion, our study shows diffusion and brain volume abnormalities in adult male and female rugby union and league players competing at an elite level. The results are in keeping with the presence of axonal injury, potentially related to repeated head impacts. Unexpected reductions in brain volume were seen in around 50% of the rugby players studied, which might indicate excess neurodegeneration in white matter tracts affected by injury. Rugby governing bodies, including the Rugby Football Union, are increasingly active collaborators in a range of head injury prevention and management projects as well as cross-sectional studies looking at brain health in retired players.^119^^,^^120^ To date these have rarely had a major focus on reporting neuroimaging changes. Further longitudinal imaging research in active and retired rugby players is needed to understand the impact of both repeated sports-related head impacts and head injuries on brain structure, and to clarify whether the abnormalities we have observed are related to an increased risk of neurodegenerative disease and impaired neurocognitive function following elite rugby participation.

## Supplementary material

Supplementary material is available at *Brain Communications* online.

## Supplementary Material

fcab133_Supplementary_Data

## Abbreviations

CTE =: chronic traumatic encephalopathy
DAI =: diffuse axonal injury
DTI =: diffusion tensor imaging
DVI =: diffuse vascular injury
FA =: anisotropy
ISOVF =: isotropic volume fraction
JD =: Jacobian Determinant
MD =: mean diffusivity
mTBI =: mild traumatic brain injury
NDI =: neurite density index
NODDI =: neurite orientation dispersion and density imaging
ODI =: orientation dispersion index
ROI =: region of interest
SWI =: susceptibility weighted imaging
TBI =: traumatic brain injury
