# Supplementary material for: White matter abnormalities in active elite adult rugby players
Source: Brain Commun. 2021 Jul 19;3(3):fcab133. doi: 10.1093/braincomms/fcab133 (PMC8381344; doi:10.1093/braincomms/fcab133)
Supplement: fcab133_Supplementary_Data [file fcab133_Supplementary_Data.docx]

| **Characteristic** | **Rugby players (*n =*44)** | **Non-Rugby Controls (*n* = 47)** | **Non-acutely injured rugby players (*n =*28)** | **Sub-acutely injured rugby players (*n =*21)** | **Non-Sport Controls (*n* = 32)** | | **Sport Controls (*n* = 15)** | **Longitudinal non-sport controls (*n = 16*)** | |
| --- | --- | --- | --- | --- | --- | --- | --- | --- | --- |
| Age, years | 25.2 ± 3.5 | 24.3 ± 3.9 | 25.8 ± 3.3 | 25.0 ± 3.8 | 25.7 ± 3.3 | | 21.5 ± 3.6* | 33.3 ± 4.0 | |
| Male sex, *n* (%) | 41 (93) | 34 (72)* | 25 (89) | 21 (100) | 19 (59)* | | 15 (100) | 9 (56) | |
| **Career details** |  |  |  |  |  | |  |  |  |
| Length of professional career, years | 5.6 ± 3.6 | NA | 6.1 ± 3.3 | 5.3 ± 3.8 | NA | | NA | NA | |
| Previous head injuries, n | 2.6 ± 2.9 | NA | 2.5 ± 2.7 | 3.0 ± 3.3 | NA | | NA | NA | |
| **Baseline paper cognitive examination** |  |  |  |  |  | |  |  |  |
| Premorbid FSIQ | 105.6 ± 5.2* | 111.5 ± 6.6 | 106.5 ± 5.0* | 102.9 ± 5.1* | 115.7 ± 4.4 | | 109.9 ± 6.7 | NA | |
| Trail Making Test A, s | 21.1 ± 6.9 | 18.9 ± 5.3 | 21.8 ± 7.9 | 20.4 ± 4.2 | 17.2 ± 3.4 | | 19.5 ± 5.9 | NA | |
| Trail Making Test B, s | 49.5 ± 17.3* | 39.7 ± 13.7 | 48.7 ± 18.0 | 48.9 ± 14.9 | 37.2 ± 11.9 | | 40.7 ± 14.6 | NA | |
| Trail Making Test B-A, s | 28.4 ± 14.1* | 20.8 ± 10.9 | 27.0 ± 12.7 | 28.5 ± 16.2 | 20.0 ± 9.4 | | 21.2 ± 11.8 | NA | |
| Stroop Colour Naming and Word Reading Composite Score, s | 23.4 ± 3.1 | 23.5 ± 3.8 | 23.4 ± 3.4 | 23.1 ± 2.4 | 22.5 ± 3.5 | | 23.6 ± 3.9 | NA | |
| Stroop Inhibition, s | 46.4 ± 8.6 | 46.9 ± 17.6 | 46.6 ± 9.2 | 45.3 ± 7.3 | 42.4 ± 6.2 | | 51.4 ± 20.1 | NA | |
| Stroop Inhibition-Switching, s | 55.5 ± 11.7 | 55.6 ± 10.2 | 54.6 ± 12.1 | 56.6 ± 11.3 | 56.0 ± 6.4 | | 55.5 ± 11.6 | NA | |
| Stroop Inhibition-Switching  verses Baseline Contrast, s | 32.0 ± 10.3 | 32.4 ± 8.2 | 31.2 ± 10.1 | 33.5 ± 10.6 | 33.5 ± 4.8 | | 31.9 ± 9.4 | NA | |
| BVMT Total Recall | 27.8 ± 4.5* | 31.8 ± 3.5 | 27.3 ± 4.6* | 28.4 ± 4.9 | 29.8 ± 4.8 | | 32.6 ± 2.7 | NA | |
| BVMT Recognition Discrimination Index | 5.8 ± 0.4 | 5.9 ± 0.2 | 5.8 ± 0.4 | 5.9 ± 0.3 | 5.8 ± 0.4 | | 6.0 ± 0.0 | NA | |
| HVLT Total Recall | 26.1 ± 4.3* | 29.5 ± 4.3 | 26.5 ± 4.4 | 24.3 ± 3.8* | 31.0 ± 1.0 | | 29.0 ± 4.9 | NA | |
| HVLT Recognition Discrimination Index | 10.6 ± 1.3 | 11.2 ± 1.1 | 10.6 ± 1.4 | 10.5 ± 0.7 | 11.6 ± 0.5 | | 11.0 ± 1.2 | NA | |
| **Baseline computerised cognitive examination** |  |  |  |  | |  |  |  |  |
| Visuospatial Working Memory | 7.8 ± 1.1 | 7.9 ± 1.1 | 7.7 ± 1.0 | 8.0 ± 0.8 | 8.0 ± 1.3 | | 7.8 ± 0.9 | NA | |
| Paired Associates | 5.3 ± 1.1 | 5.2 ± 1.2 | 5.3 ± 1.1 | 5.3 ± 0.9 | 5.1 ± 1.3 | | 5.3 ± 1.0 | NA | |
| Self Ordered Search | 8.8 ± 1.4 | 8.6 ± 1.9 | 8.7 ± 1.5 | 8.5 ± 1.3 | 8.4 ± 2.1 | | 8.9 ± 1.7 | NA | |
| Feature Match | 118.0 ± 23.4 | 119.7 ± 34.6 | 118.8 ± 24.2 | 110.2 ± 25.1 | 121.6 ± 37.1 | | 116.9 ± 31.6 | NA | |
| Odd One Out | 14.8 ± 2.0 | 15.4 ± 2.0 | 14.7 ± 2.1 | 15.3 ± 1.9 | 15.9 ± 1.8 | | 14.8 ± 2.1 | NA | |
| Spatial Planning | 16.9 ± 5.3 | 18.3 ± 6.1 | 16.9 ± 5.7 | 16.8 ± 4.6 | 19.7 ± 6.6 | | 16.3 ± 4.6 | NA | |
| *Fractals task* |  |  |  |  |  | |  |  |  |
| Identification error - 1 item | 99.2 ± 2.7 | 99.7 ± 1.7 | 99.2 ± 2.7 | 99.4 ± 2.5 | 100.0 ± 0.0 | | 99.3 ± 2.6 | NA | |
| Identification error - 3 item | 91.2 ± 5.3 | 93.2 ± 5.0 | 91.1 ± 5.2 | 91.4 ± 5.5 | 92.5 ± 5.4 | | 94.1 ± 4.3 | NA | |
| Localisation error - 3 item | 6.6 ± 1.7 | 6.2 ± 2.2 | 6.6 ± 1.8 | 6.7 ± 1.5 | 6.5 ± 2.8 | | 5.9 ± 1.2 | NA | |
| Swap error (4s delay) | 13.3 ± 9.3 | 14.9 ± 9.3 | 13.2 ± 8.9 | 12.2 ± 9.5 | 15.6 ± 9.6 | | 14.0 ± 9.1 | NA | |
| **Baseline MRI characteristics** |  |  |  |  |  | |  |  |  |
| DTI abnormalities, *n* (%) | 7 (17) | 1 (2) | 5 (19) | 2 (10) | 1 (3) | | 0 (0) | NA | |
| Microhaemorrhages, *n* (%) | 3 (7) | 1 (2) | 1 (4) | 2 (10) | 0 (0) | | 1 (7) | NA | |
| **Demographic characteristics** |  |  |  |  |  | |  |  |  |
| Weight, kg |  |  | 99 ± 15 | 102 ± 11 | NA | | 80 ± 7 | NA |  |
| Height, cm |  |  | 184 ± 9 | 185 ± 8 | NA | | 184 ± 9 | NA |  |
| Alcohol intake, units per week | 4.6 ± 5.9 | 6.2 ± 5.8 | 5.5 ± 5.0 | 4.4 ± 6.3 | 6.12 ± 5.5 | | 6.3 ± 6.6 | NA |  |
| **Head injury details** |  |  |  |  |  | |  |  |  |
| Time since injury, days |  | NA | NA | 4.7 ± 1.2 | NA | | NA | NA |  |
| Symptom Severity Score at time of injury | NA | NA | NA | 10.3 ± 5.3 | NA | | NA | NA |  |
| Return to play duration, days | NA | NA | NA | 7.2 ± 1.5 | NA | | NA | NA |  |

**Supplementary Table 1. Participant demographics and neuropsychological test performance.**

* Significance at *P* < 0.05, uncorrected. Comparisons shown were either statistical tests between Rugby players and Non-Rugby Controls (independent t-tests or Chi-Squared tests) or a comparison of means in acutely injured rubgy players, non-acutely injured rugby players, Non-Sport Controls, and Sport Controls using an ANOVA. Significant results indicated were Tukey post hoc tests relative to Non-Sport Controls. FSIQ = Full Scale Intelligence Quotient, BVMT = Brief Visuospatial Memory Test, HVLT = Hopkins Verbal Learning Test.
